# Establishment of *Dittrichia viscosa* L. Hairy Roots and Improvement of Bioactive Compound Production

**DOI:** 10.3390/plants13223236

**Published:** 2024-11-18

**Authors:** Annalisa Paradiso, Miriana Durante, Sofia Caretto, Angelo De Paolis

**Affiliations:** Istituto di Scienze delle Produzioni Alimentari (ISPA), Consiglio Nazionale delle Ricerche (CNR), Via Monteroni, 73100 Lecce, Italy; annalisa.paradiso@cnr.it (A.P.); miriana.durante@ispa.cnr.it (M.D.)

**Keywords:** *Dittrichia viscosa*, hairy root cultures, phenolics, flavonoids, antioxidant activity

## Abstract

*Dittrichia viscosa* is a ruderal plant species growing along roadsides and well adapting to extreme environmental conditions. *D. viscosa* plant tissues, especially leaves, are known to be a rich source of bioactive metabolites which have antioxidant, cytotoxic, antiproliferative and anticancer properties. Hairy root cultures are a suitable biotechnological system for investigating plant metabolic pathways and producing specialized metabolites in in vitro conditions. In this study, *D. viscosa* hairy root transformed lines induced by *Agrobacterium rhizogenes* ATCC15834 were obtained using leaf explants, and the integration of *rolB* and *rolC* genes in the genomes of transformed hairy roots were confirmed by PCR analysis. Three hairy root *D. viscosa* lines (DvHrT1, DvHrT4 and DvHrT5) having different phenotypic features were characterized in terms of total phenolics, flavonoids and antioxidant activity. Correlated with antioxidant activity, phenolic and flavonoid content of DvHrT1 was significantly higher than control roots and the other DvHrT lines. Our results suggest that *D. viscosa* hairy roots can be a valuable tool for producing various bioactive compounds having antioxidant activity and are to be further investigated to produce other specific molecules that could find application in agricultural or pharmaceutical fields.

## 1. Introduction

*Dittrichia viscosa* is a ruderal species, growing along roadsides of the Mediterranean region and well adapting to extreme environmental conditions. It is a perennial herbaceous plant, belonging to the Asteraceae family. The life cycle of *D. viscosa* begins in March and ends with full flowering, with typical yellow flowers, in the months of October–November. The surface of plant organs, mainly leaves, has many glandular hairs secreting a sticky resin with a distinctive smell. *D. viscosa* tissues are a rich source of potential bioactive compounds which could be used in agricultural and pharmaceutical applications [1,2]; its components present a broad range of biological activities, such as anti-inflammatory, antifungal, antiulcer, antiviral and phytotoxic properties. On the other hand, *D. viscosa* has been largely utilized in folk medicine for treating several diseases including diabetes, respiratory disorders and cancer [3,4,5].

Many bioactive metabolites were extracted from plant organs including leaves, stems, roots and flowers, and although in many cases, they are useful for the plants, in most cases, they are not needed for plant growth, development and reproduction [6]. Plant specialized metabolites are natural chemical compounds used by humans in various industries, such as nutraceutical and cosmetic industries, and in agricultural control, as well as pharmaceutical applications. Due to their contents often low in plants, biotechnological methods should be developed to obtain large-scale production of specific metabolites [7]. In vitro plant tissue cultures easily provide good sources for the production of specialized compounds in higher qualities and quantities [8,9,10]. In the effective tissue culture systems for biotechnological applications, callus and suspension cell cultures, hairy root cultures and micropropagation approaches are included [11,12]. Hairy root cultures are effective biotechnological systems both for studying plant metabolic pathways and obtaining specialized metabolites [13,14]. It is well known that the hairy roots induced by *Agrobacterium rhizogenes* represent an attractive biological system known for rapid root growth and high genetic and biosynthetic stability. In numerous studies, the hairy root cultures of several officinal plant species were reported to enhance in vitro phytochemical production [11,12,15]. This is the case of *Ocimum basilicum* producing rosmarinic acid, known for its antimicrobial and anti-inflammatory activities [16], *Salvia plebeia* producing phenolic compounds with antioxidant activity [17] or *Panax ginseng* producing ginsenosides, which have several pharmacological activities such as anti-inflammatory, anticancer and antiaging [18].

Until now, despite the interest in the identification and characterization of phytochemical compounds from *D. viscosa* plants [19,20], no reports have been published on in vitro hairy root cultures of this species induced by *A. rhizogenes* and their possible application for the production of industrially important metabolites.

This study was conducted with the aim of obtaining hairy roots of *D. viscosa* by *A. rhizogenes* infection and exploring their capacity of bioactive compound production. For this purpose, *D. viscosa* hairy roots were induced using *A. rhizogenes* ATCC15834 strain and characterized in terms of phenolics, flavonoids and antioxidant activity.

## 2. Results

### 2.1. Induction and In Vitro Culture of Hairy Roots

Hairy roots from *D. viscosa* leaf explants were induced by *A. rhizogenes* ATCC15834. The highest percentage of root formation (97%) was observed in leaf explants from 30-day-old in vitro grown plants, using half-strength Murashige and Skoog (½ MS) as cultivation medium [21].

**Figure 1:**
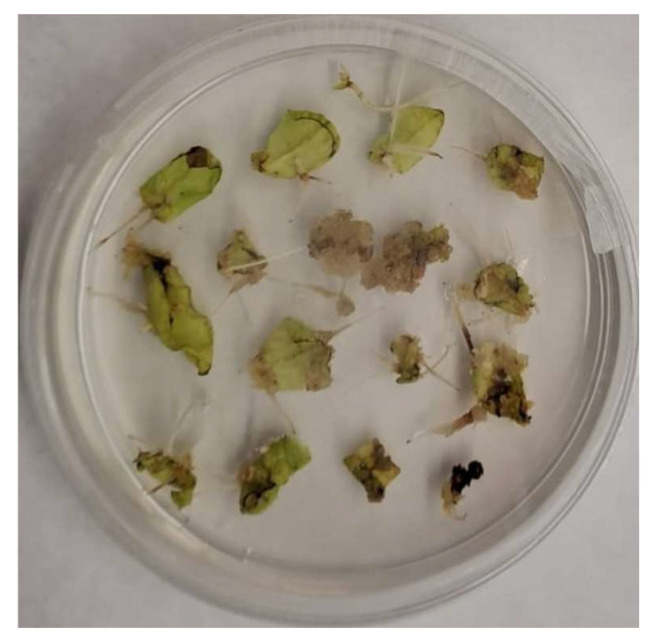
Hairy root initiation in *Dittrichia viscosa* leaf explants 15 days after infection with *Agrobacterium rhizogenes* ATCC15835.

**Figure 2:**
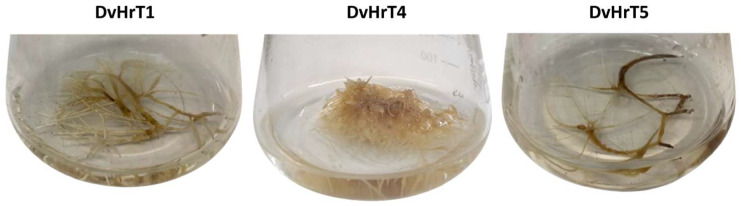
Hairy root cultures of *Dittrichia viscosa* after 4 weeks of growth in 250 mL flask containing 50 mL of liquid medium. Representative images of DvHrT1, DvHrT4 and DvHrT5 indicating different morphological features.

No hairy roots were observed in untransformed leaf explants that were used as experimental control. Hairy roots appeared 15 days after the infection at the wound sites of *D. viscosa* leaf tissues (Figure 1). Out of a total of 50 leaf explants used in the experiment, 48 showed the development of hairy root tissues. Plant stems and internode tissues were also used, but no hairy roots were observed. Five hairy root lines (DvHrT1-5) deriving from independent events of transformation were propagated separately, and three (DvHrT1, DvHrT4 and DvHrT5) showing a different phenotype (Figure 2) in terms of number of root branches and growth rate (0.256 g/week, 0.754 g/week and 0.125 g/week for DvHrT1, DvHrT4 and DvHrT5, respectively) were chosen for further characterization. Moreover, the spent medium of one-month-old DvHrT lines appeared differently brownish, possibly due to differences in the production and release into the growth medium of some specific metabolites.

### 2.2. Confirmation of Integration of the rolB and rolC Genes in the DvHrT Lines

**Figure 3:**
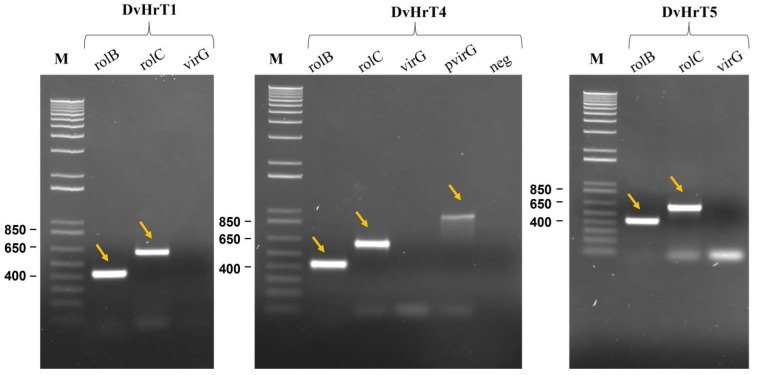
PCR analysis of *Dittrichia viscosa* hairy roots. Detection of *rolB* (423 bp), *rolC* (626 bp) and *VirG* (965 bp) genes in DvHrT1, DvHrT4 and DvHrT5 hairy roots by PCR analysis. M, marker; *pvir*G, plasmid DNA from *Agrobacterium rhizogenes* ATCC15835. Arrows indicate the amplified fragments.

The Ri T-DNA region of the *A. rhizogenes* Ri plasmid is responsible for the hairy root induction in many dicotyledon plant species. Although all *rol* genes contribute to the hairy root induction [22], *rolB* overexpression induced many hairy roots in transgenic plants, revealing to have a crucial role in pathogenicity. The integration of *rolB* and *rolC* genes in the genome of DvHrT lines was demonstrated by PCR amplification of a portion of the *rolB* and *rolC* genes and a portion of the *VirG* gene that contributes to the HR induction but is not transferred to the plant. Using genomic DNA isolated from Dv HRT lines as a template, fragments of 423 bp and 626 bp for *rolB* and *rolC* genes, respectively, were amplified. As expected, no amplification of the *virG* gene was observed using DvHr line genomic DNA, while an amplified fragment of 965 bp was detected in pRiA4 plasmid DNA of the *A. rhizogenes* 15834 strain (Figure 3). These results demonstrated that the Ri T-DNA region of *A. rhizogenes* 15834 was integrated into the genome of the putative *D. viscosa* hairy roots, and they confirmed that the induced roots were indeed hairy roots.

### 2.3. Total Phenolic and Flavonoid Content in DvHrT Lines

**Figure 4:**
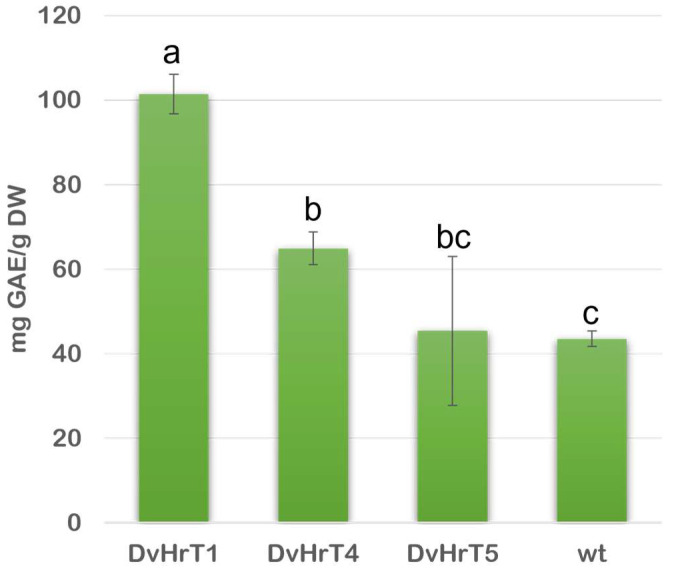
Total phenolic content (TPC) of *Dittrichia viscosa* hairy root lines compared to wt roots was expressed as gallic acid equivalent (GAE/DW). The data indicate mean ± SD from three independent experiments. Different letters indicate significant differences at *p* < 0.05 (Tukey test).

Total phenolic (TPC) and flavonoid content (TFC) were measured in DvHrT1, DvHrT4 and DvHrT5 lines compared to wild-type roots. The TPC values, reported as mg of gallic acid equivalent/g dried powder, were higher in DvHrT1 and DvHrT4 than wild-type Dv roots (+134% and +51%, respectively); the increase observed for DvHrT1 was also significant. On the contrary, the DvHrT5 line showed a level comparable to wild-type roots (Figure 4).

**Figure 5:**
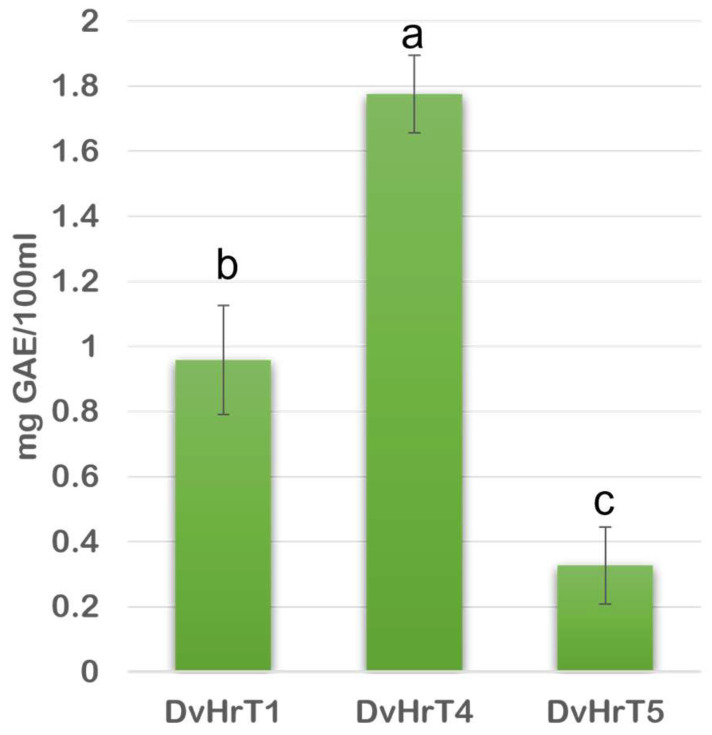
Total phenolic content (TPC) in liquid medium of *Dittrichia viscosa* hairy root lines was expressed as gallic acid equivalent (GAE/100 mL). The data indicate mean ± SD from three independent experiments. Different letters indicate significant differences at *p* < 0.05 (Tukey test).

Since it is known that hairy root cultures induced by *A. rhizogenes* are an effective system for studying the possible exudation of specific metabolites [23], TPC was also analyzed in the culture spent medium. Although DvHrT4 had a lower intracellular level of TPC than DvHrT1, it accumulated a higher TPC level in the spent medium, likely due to the higher fresh weight. Indeed, TPC accumulation resulted higher in the medium of this line (1.775 ± 0.119 mg GAE/100 mL) compared to the DvHrT1 line (0.958 ± 0.168 mg GAE/100 mL) and the DvHrT5 line (0.327 ± 0.119 mg GAE/100 mL) (Figure 5).

**Figure 6:**
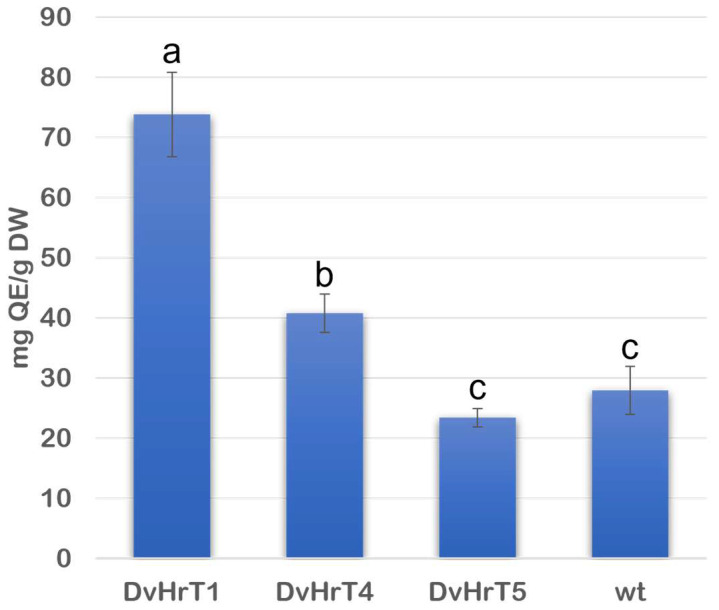
Total flavonoid content (TFC) of *Dittrichia viscosa* hairy root lines compared to wt roots was expressed as quercetin equivalent (QE/DW). The data indicate mean ± SD from three independent experiments. Different letters indicate significant differences at *p* < 0.05 (Tukey test).

Additionally, total flavonoid content (TFC) expressed as mg of quercetin equivalent/g dried powder was analyzed in DvHrT1, DvHrT4 and DvHrT5 lines compared to wt roots. Coherently with TPC, the DvHrT1 showed the highest level, being 164% higher than wild-type Dv roots. Also, DvHrT4 showed a significant increase of 45% compared to control, whereas in DvHrT5, flavonoid content remained low and comparable to Dv wild-type roots (Figure 6).

### 2.4. HPLC Analysis of Phenolic Compounds

**Figure 7:**
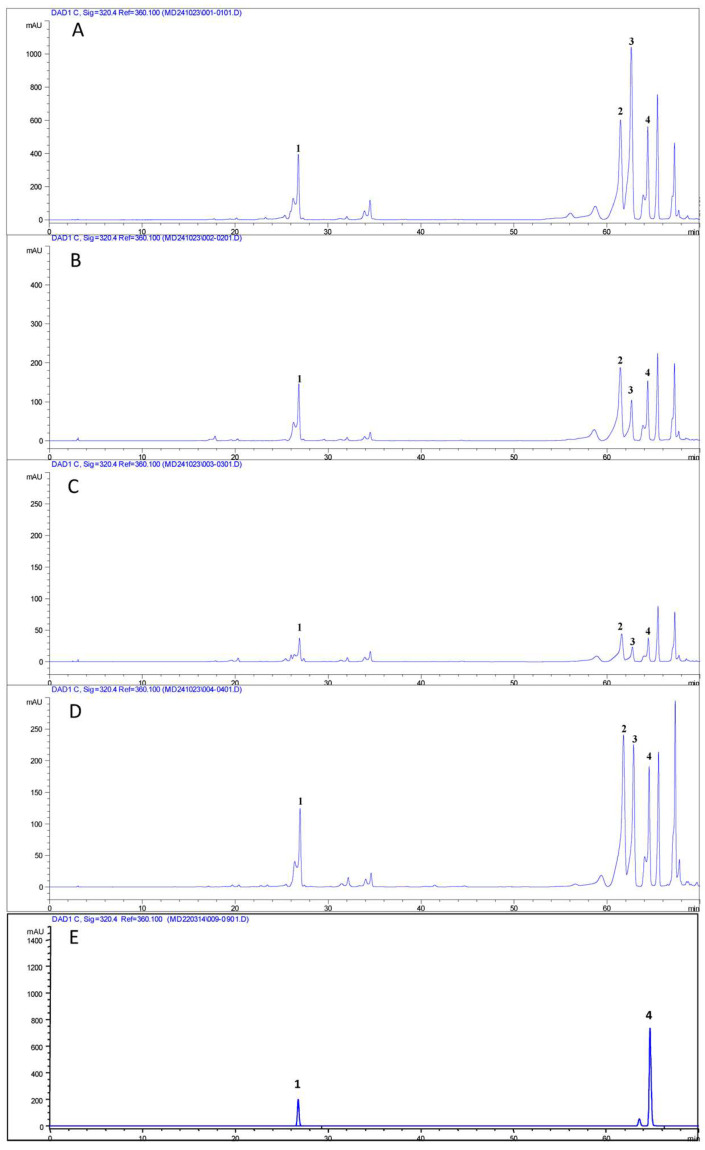
HPLC chromatograms of methanolic extracts of *Dittrichia viscosa* hairy root lines and wild-type roots. (**A**): DvHrT1; (**B**): DvHrT4; (**C**): DvHrT5; (**D**): wt roots; (**E**): chlorogenic acid and rosmarinic acid standards. Peak 1: chlorogenic acid; peak 2: di-O-caffeoylquinic acid; peak 3: di-O-caffeoylquinic acid isomer; peak 4: rosmarinic acid.

HPLC analysis of a methanolic extract was performed to compare the phenolic profile of hairy root lines and wt roots (Table 1). In each analyzed sample, four phenolic acids, namely chlorogenic acid, di-O-caffeoylquinic acid, di-O-caffeoylquinic acid isomer and rosmarinic acid, were detected (Figure 7), as similarly reported in leaf extracts of *D. viscosa* [24,25]. In DvHrT lines, the most abundant compound was represented by di-O-caffeoylquinic acid, followed by rosmarinic acid. Chlorogenic acid had the lowest concentration in all DvHrT lines. Differently, in wt roots, the two di-O-caffeoylquinic acids were the most abundant phenolic acids, with chlorogenic acid being the least. Statistical analysis showed that chlorogenic acid, di-O-caffeoylquinic acid and rosmarinic acid contents increased significantly in DvHrT1 and DvHrT4 compared to wt roots. Only di-O-caffeoylquinic acid isomer showed higher values in wt roots compared to DvHrT lines. In the DvHrT5 line, all phenolic acids had lower levels compared to DvHrT1, DvHrT4 lines and wt roots.

### 2.5. In Vitro Antioxidant Assay

**Figure 8:**
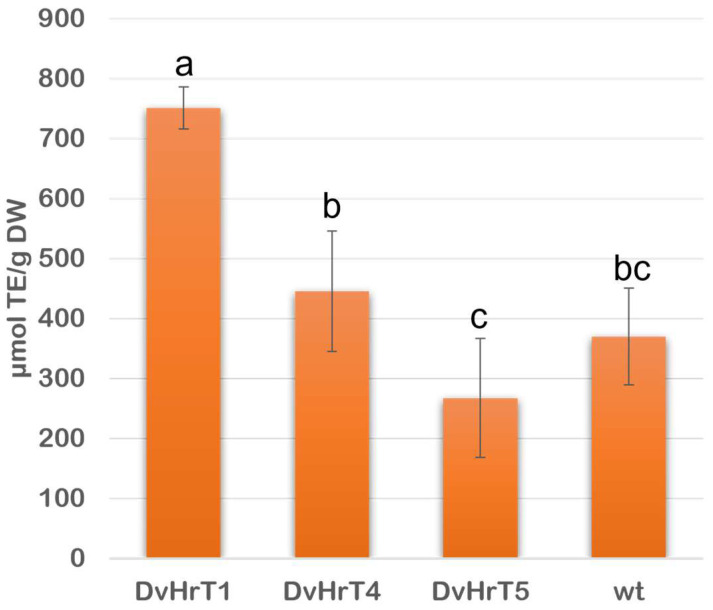
Antioxidant activity of *Dittrichia viscosa* hairy root lines compared to wt roots expressed as Trolox equivalent (TE/DW). The data indicate mean ± SD from three independent experiments. Different letters indicate significant differences at *p* < 0.05 (Tukey test).

ABTS assay was used to evaluate the antioxidant activity of *D. viscosa* hairy root lines compared to wt roots (Figure 8). The three hairy root lines differed from each other, with DvHrT1 having the highest values and being significantly higher than the other two hairy root lines and wt roots. DvHrT4 and DvHrT5 did not show significant differences compared to wt roots.

## 3. Discussion

Hairy roots obtained by *A. rhizogenes* are extensively used for the investigation of plant metabolism and the large-scale production of specialized metabolites, since biosynthetic pathways in hairy roots work in a similar manner or even better than those of original plant roots or those of whole plants [26,27].

In the present study, for the first time, *D. viscosa* hairy roots were obtained by infection with the *A. rhizogenes* strain ATC 15834 using leaf explants from in vitro grown plants. The obtained DvHRT lines had a different phenotype, likely due to different T-DNA insertion sites. During the Agrobacterium tissue infection, the bacterial T-DNA region is incorporated into the plant genome [28]. The plant genome site of integration, as well as the contact with pathogenic bacteria, may affect some parameters characterizing “hairy” roots [11]. The *rolB* gene has a tyrosine phosphatase activity [26] likely implicated in signal transduction processes associated with auxin concentration and/or sensitivity; it represents the key gene for initiating and elongating hairy roots [21]. The various and tricky effects of *rolB* on transformed plant cells may be due to the alterations caused by this gene in RNA silencing occurrence by microRNA overexpression [27]. In various plant species, such as *Withania somnifera* [29], *Ajuga bracteosa* [30], *Dendranthema grandiflora* [31] and *Solanum tuberosum* [32], differing phenotypes were reported for hairy roots induced by *A. rhizogenes*. In our study, hairy roots from independent transformation events were separately grown, showing different phenotypes. DvHrT4 lines showed a rapid development of white fragile hairy root tissue, in which the presence of the *rolB* and *rolC* genes was confirmed. The DvHrT1 and DvHrT5, although positive to the presence of *rolB* and *rolC* genes, showed a slower growth and brown coloration of the tissues. To sum up, the molecular mechanism of hairy root differentiation is unclear, but it is possible that the genome insertion site of *rol* genes, as well as the complex effects of these genes in transformed plant cells, influencing the RNA silencing pathways [33] could be a possible cause of the different DvHrT phenotypes.

At present, hairy root cultures of a huge number of plant angiosperm species (more than 400) have been established and applied for various purposes [14,15,34]. Hairy root induction was influenced by many variables, such as plant species, explant kind, bacterial strain and concentration, infection technique and co-cultivation protocols. Hairy root cultures are characterized by high proliferation rates in the absence of hormone administration, lack of geotropism and lateral branching; they also have genetic stability. Being stable and highly productive, hairy root cultures have been studied for many years because of the capability to produce bioactive compounds synthesized in wild-type roots, although in small quantities.

In this study, we show that the obtained DvHrT lines accumulated antioxidant compounds such as phenolics and flavonoids. Moreover, different lines of *D. viscosa* hairy roots exhibited different levels of antioxidant metabolites, with one line, DvHrT1, significantly higher compared to other lines and non-transformed roots. Particularly, phenolic acids, such as chlorogenic, di-O-caffeoylquinic and rosmarinic acids, identified by HPLC analysis, were more abundant in DvHrT1 and DvHrT4 compared to wt roots. Interestingly, phenolic compounds, such as di-O-caffeoylquinic acid, due to the presence of functional groups (hydroxyl and caffeoyl groups), seem to be responsible for a remarkable antioxidant capacity to scavenge reactive oxygen species in *D. viscosa* tissues [24,35]. Accordingly, the highest level of antioxidant activity in DvHrT1 could be due the significant increase in di-O-caffeoylquinic acid observed in this line. More generally, antioxidant activity detected in the methanolic extracts of the three DvHrT lines well correlated with TPC and TFC. Our results are also in agreement with previous studies that investigated hairy roots established from different plant species. Hairy root cultures of *Dracocephalum moldavica* [36], *Solanum trilobatum* [37], *Momordica dioica* [38] and *Cucumis anguria* [39] reported enhanced values of TPC and TFC and had stronger antioxidant activities compared to the roots of field-grown plants. Moreover, hairy root cultures of *Lactuca serriola* had improved levels of phenolics and flavonoids and showed a 31.6–50% increase in DPPH values compared to wild-type roots [40].

Overall, the successful establishment of *D. viscosa* hairy roots producing antioxidant metabolites, including phenolics and flavonoids, can be a valuable tool in a biotechnological perspective for producing various bioactive compounds for possible nutraceutical applications. Elicitation strategies will help improve the in vitro production system by optimizing the performance of the selected DvHrT lines. On the other hand, *D. viscosa* hairy root cultures, due to the rich metabolism of this species, could be further investigated for the production of other bioactive molecules for agricultural and pharmaceutical applications.

## 4. Materials and Methods

### 4.1. Plant Material and Culture Medium Preparation

Mature seeds of *D. viscosa* collected from wild plants grown near the Unisalento campus (40°20′05.5″ N latitude; 18°07′16.5″ E longitude) were recovered and used for seedling establishment (October/November). Murashige and Skoog (MS) (Duchefa Biochemie, Haarlem, The Netherlands) [21] basal medium was used for the in vitro germination and establishment of aseptic seedlings. The full-strength MS basal medium contained 3% (*w*/*v*) sucrose and 0.7% (*w*/*v*) agar as solidifying agent without plant growth regulators. The pH was adjusted to 5.7 prior to the addition of agar (Duchefa Biochemie, Haarlem, The Netherlands). The medium was autoclaved at 121 °C and 1.06 kg/cm^2^ for 20 min and used to prepare Petri plates with 25 mL each.

### 4.2. Seed Sterilization and In Vitro Germination

Seeds of *D. viscosa* were immersed in 70% ethanol (Sigma-Aldrich, Milan, Italy) for 1 min, followed by 30% sodium hypochlorite for 15 min, with some drops of Tween 20 (Sigma-Aldrich, Milan, Italy) as a wetting agent. The seeds were rinsed five times in sterile distilled water to remove all traces of the sterilant, and seeds were cultured on solid MS Petri plates and incubated at 25 ± 1 °C under 16 h/8 h photoperiod using cool white fluorescent light. Plants of *D. viscosa* were grown in MS basal medium at 25 °C in a 16 h light/8 h dark photoperiod and micropropagated monthly by tip shoots.

### 4.3. Preparation of A. rhizogenes Strains

The *A. rhizogenes* strain ATCC15834 was used in the genetic transformation experiments. A single colony of the bacterial strain was grown in TY liquid medium (0.5% Tryptone, 0.3% yeast extract, 0.09% CaCl_2_·2 H_2_O pH 6.8) containing 50 mg/l rifampicin (Duchefa Biochemie, Haarlem, The Netherlands), at 28 °C, 120 rpm on a shaker incubator. The bacterial suspension (OD600 = 0.6) was centrifugated at 3000 rpm per 15 min; the pellet was resuspended in ½ MS medium and used for infection of leaf explants.

### 4.4. Induction and Cultivation of Hairy Roots

Hairy roots were induced from leaf explants of *D. viscosa* (1 cm) and infected with the *A. rhizogenes* strain ATCC15834. The tissue explants were treated with a bacterial suspension for 15 min, dried on sterilized paper and incubated on ½ MS solid medium for 48 h. After the co-cultivation, the explants were transferred to MS solid medium supplemented with 500 mg/L cefotaxime (Duchefa Biochemie, Haarlem, The Netherlands). Subsequently, explants were subcultured on MS solid medium at one-week intervals using 400, 300, 200, 100 and 0 mg/L cefotaxime. Finally, 1–2 cm lengths of the hairy roots were transferred in 50 mL MS liquid medium in 250 mL flash and incubated for 4 weeks in the dark at 25 °C and 100 rpm. Five randomly chosen hairy root (DvHrT1-T5) samples derived from independent transformation events were propagated separately.

### 4.5. DNA Extraction and PCR Analysis of Hairy Roots

Genomic DNA was extracted from 100 mg of fresh hairy root lines (DvHr1, DvHrT4 and DvHrT5) according to the CTAB method. DNA (50–100 ng) was used as a template for PCR amplification to detect the *rol*B, *rol*C and *VirG* genes using specific primers purchased from Thermo Fisher Scientific, Waltham, MA, USA (Table 2). Plasmid DNA extracted from ATC15834 was used as a positive control, while genomic DNA of non-transformed wild-type plants was used as a negative control. The PCR conditions included an initial denaturation at 94 °C for 3 min; 35 cycles at 94 °C for 20 s, 60 °C for 20 s and 72 °C for 30 s; and a final extension at 72 °C for 10 min. The PCR products were separated on 1.5% TAE agarose gel and visualized with a ChemiDoc MP Imaging System (BIO-RAD, Hercules, CA, USA) gel documentation system.

### 4.6. Establishment of the Hairy Roots Culture

Thirty-day-old hairy roots were cut in pieces (about 1.5 cm each) and transferred to a 250 mL flask containing 50 mL of liquid ½ MS basal medium and incubated in a growth chamber at 25 ± 2 °C in agitation (100 rpm) in the dark. The hairy roots were subcultured every 4 weeks and used for further analysis.

HR and root tissues were frozen in liquid nitrogen, lyophilized (Labconco, Kansas City, MO, USA), reduced to a powder and stored at −20 °C until analysis.

### 4.7. Extraction Method

The extracts used for the estimation of the phenolic compound, flavonoid content and antioxidant capacity were obtained according to Mottaki et al. [41] with some modifications. All reagents were purchased from Sigma-Aldrich (Milan, Italy).

Briefly, in 15 mL Falcon tubes, ≈50 mg of each lyophilized sample was weighed and, subsequently, 80% methanol (1:100; *w*/*v*) was added. The mixture was vortexed, treated with ultrasonic wave for 5 min and centrifuged (Beckman Coulter Allegra X-15R) for 15 min at 4500× *g* at 4 °C.

### 4.8. Phenolic Compound Analysis

Diluted extract (1:10; *v*:*v*) was mixed with 0.5 mL of 10% Folin–Ciocalteu reagent (Sigma-Aldrich, Milan, Italy); after 10 min of incubation in dark and RT conditions, 0.5 mL of 7% NaCO_3_ and 0.15 mL of H_2_O were added. After 90 min of incubation at RT, in dark conditions, absorbance at 760 nm was read by means of a multiplate reader (Infinite M200 Tecan, Männedorf, Switzerland). Gallic acid (in 1–150 µg/mL range) was used for calibration curve.

An aliquot of culture medium from the different DvHRT lines was centrifuged (4500× *g* for 10 min at RT), and a volume of 0.1 mL was used for the analysis of TPC in the culture medium. An equal volume of fresh medium was used as a blank.

### 4.9. Flavonoid Analysis

The flavonoid content was determined according to Mottaki et al. [41] with slight modifications. Briefly, 0.1 methanolic extract (methanol in blank sample) was placed in a 2 mL Eppendorf tube. For each sample, 0.4 mL of 70% methanol and 30 µL of 5% NaNO_2_ solution were added. After incubation at RT for 5 min, 30 µL of 10% AlCl_3_ solution was added, and the mixture was incubated for another 5 min. Then, 0.2 mL of 1 M NaOH was added, and the total volume was increased to 2 mL using a 70% methanol solution. Samples were mixed and allowed to stand for 30 min at 35 °C. The absorbance was read at 420 nm, and flavonoid contents were expressed as mg quercetin equivalents (QEs) per dry mass.

### 4.10. HPLC Analysis of Soluble Phenols

Aliquots (50 mg) of lyophilized powder of DvHrT and wt root samples were extracted with 2 mL of aqueous methanol (80%, *v*/*v*). Samples were sonicated in a water bath for 30 min and incubated under magnetic stirring for 5 h. After centrifugation at 4500 g for 10 min (4 °C), supernatants obtained were used for HPLC determination using an Agilent 1100 Series HPLC system (Agilent Technologies, Santa Clara, CA, USA) equipped with a Phenomenex-luna 5 µm C18 (2) 100 Å column (250 × 4.6 mm), (Phenomenex, Torrance, CA, USA). For identification and quantification of chlorogenic acid and rosmarinic acid, standards (Sigma-Aldrich, Milan, Italy) were used. For di-O-caffeoylquinic acid and di-O-caffeoylquinic acid isomer, peaks were identified by comparing DvHrT and wt root chromatographic profiles with profiles of *D. viscosa* leaf extracts previously characterized and confirmed by NMR, as reported by Anglana et al. [25]; quantification was obtained using caffeic acid standard (Sigma-Aldrich, Milan, Italy).

### 4.11. Antioxidant Activity

Trolox equivalent antioxidant activity was analyzed according to Tonto et al. [42] with modification. Briefly, ABTS (Sigma-Aldrich, Milan, Italy) stock solution was prepared, dissolving 7 mM of ABTS solution in 2.45 mM of potassium persulfate. After incubation in the dark for 16 h, ABTS stock solution was diluted with phosphate buffer of 0.05 mM at pH = 7.4 to obtain an absorbance of 0.7 ± 0.02 at 730 nm. An amount of 20 µL of the test sample or Trolox standard was placed in a 96-well microplate, after which 180 µL of radical ABTS was added. After 30 min of incubation, the absorbance was measured at 730 nm by means of a microplate reader (Infinite M200 Tecan, Männedorf, Switzerland). Aqueous solutions of Trolox concentrations (20–200 µM) were used for calibration.

## Figures and Tables

**Table 1:** HPLC analysis of phenolic compounds of *Dittrichia viscosa* hairy root lines compared to wt roots; values were expressed as mg/g DW. The data indicate mean ± SD from three independent experiments. Different letters on the same line indicate significant differences at *p* < 0.05 (Tukey test).

Phenolic Compounds	DvHrT1	DvHrT4	DvHrT5	Wt
chlorogenic acid	2.653 ± 0.212 a	1.156 ± 0.123 b	0.454 ± 0.047 d	0.623 ± 0.0747 c
di-O-caffeoylquinic acid	22.449 ± 2.402 a	8.001 ± 0.960 b	2.424 ± 0.315 d	6.541 ± 0.589 c
di-O-caffeoylquinic acid isomer	3.021 ± 0.241 b	3.111 ± 0.279 b	1.212 ± 0.157 c	4.360 ± 0.422 a
rosmarinic acid	12.245 ± 1.567 a	3.556 ± 0.427 b	1.818 ± 0.215 d	2.492 ± 0.263 c

**Table 2:** Primers used and amplification products of *rolB*, *rolC* and *virG* genes from DNA of *Dittrichia viscosa* hairy root cultures.

Primer	Sequence	Fragment Length
*rol*B F	5′-GCTCTTGCAGTGCTAGATTT-3′	423 bp
*rol*B R	5′-GAAGGTGCAAGCTACCTCTC-3′	
*rol*C F	5′-GCTCTTGCAGTGCTAGATTT-3′	626 bp
*rol*C R	5′-GAAGGTGCAAGCTACCTCTC-3′	
*vir*G F	5′-GCTCTTGCAGTGCTAGATTT-3′	965 bp
*vir*G R	5′-GAAGGTGCAAGCTACCTCTC-3′	

## Data Availability

All data are included in the article.
